# Left ventricular remodeling in rheumatoid arthritis patients without clinical heart failure

**DOI:** 10.1186/s13075-023-03113-8

**Published:** 2023-07-21

**Authors:** Elizabeth Park, Kazato Ito, Christopher Depender, Jon T. Giles, Joan Bathon

**Affiliations:** 1 Division of Rheumatology, Columbia University Vagelos College of Physicians and Surgeons and New York Presbyterian Hospital, 630 W 168Th St, P&S 3-450, New York, NY 10032 USA; 2 Division of Cardiology, Columbia University Vagelos College of Physicians and Surgeons and New York Presbyterian Hospital, New York, NY USA

**Keywords:** Rheumatoid arthritis, Heart failure, Left ventricular remodeling, Interleukin-6

## Abstract

Rheumatoid arthritis (RA) patients have a 1.5- to twofold higher risk of developing heart failure (HF) and a twofold increased risk of HF-associated mortality compared to those without RA. HF is preceded subclinically by left ventricular (LV) remodeling in the general population. There is a relative absence of prospective studies following RA patients from pre-clinical to clinical HF as well as prospective studies of LV remodeling in RA without clinical HF. In our study, 158 RA patients without clinical HF were enrolled and underwent transthoracic echocardiography (TTE) at baseline and on follow-up between 4 and 6 years. Extensive characterization of RA disease activity and cardiovascular risk factors were performed. LV remodeling was prevalent at 40% at baseline and increased to 60% over time. Higher levels of interleukin-6 (IL 6) were associated with concentric LV remodeling on follow-up. The use of tocilizumab was also significantly associated with baseline LV remodeling (relative wall thickness). These findings suggest a role for IL-6 as a biomarker for LV remodeling in RA patients without clinical HF. Future research should focus on prospective follow-up of LV remodeling and the effects of IL-6 inhibition on LV remodeling in RA patients.

**Supplementary Information:**

The online version contains supplementary material available at 10.1186/s13075-023-03113-8.

## Introduction

People with rheumatoid arthritis (RA) have a 1.5- to twofold higher risk of developing heart failure (HF) and a twofold increased risk of HF-associated mortality compared with people without RA. Emerging data suggest that the dominant clinical HF phenotype in RA appears to be HF with preserved ejection fraction (HFpEF) [1]. In the general population, HFpEF is thought to be preceded subclinically by the development of LV diastolic dysfunction and concentric remodeling, while HF with reduced ejection fraction (HFrEF) is more typically preceded by systolic dysfunction and eccentric remodeling [2–5]. While there are no prospective studies that have followed RA patients from pre-symptomatic phase to clinical HF, numerous reports confirm that RA patients without clinical HF have a higher rate of subclinical diastolic dysfunction compared to age- and gender-matched controls [6]. However, reports on LV remodeling and its determinants in RA patients without clinical HF are few. Several cross-sectional studies [7–9] confirm a higher prevalence of concentric hypertrophy and remodeling in RA vs age- and gender-matched non-RA controls. However, there are few data to date on the rate of progression of subclinical LV remodeling and its determinants. We evaluated not only the baseline prevalence of LV remodeling but also its progression, in an RA cohort without clinical HF. Furthermore, we hypothesized that measures of RA disease activity would convey risk for baseline and/or progression of LV concentric remodeling.

## Methods

The RHYTHM cohort (*n* = 158) was designed as a cross-sectional study in 2011 to identify myocardial phenotypes in RA patients without clinical cardiovascular disease (CVD). Patients were subsequently re-recruited for a follow-up visit 4–6 years later to investigate changes in LV structure and function over time; 60 participants completed the follow-up visit. Detailed inclusion/exclusion criteria have been published previously [10]. The baseline visit consisted of clinical questionnaires, joint examination, biospecimen collection, echocardiography, and cardiac PET-CT scan. Echocardiography was repeated at follow-up. The study was approved by the Columbia University Irving Medical Center Institutional Review Board. All subjects provided written informed consent prior to enrollment.

### Clinical characteristics

#### Demographic and cardiovascular characteristics

The assessment of demographics, lifestyle characteristics, CV risk factors, and medications was performed as previously described [10]. Forty-four joints were examined for swelling and tenderness by the same trained joint assessor. RA disease activity was calculated with the Disease Activity Score for 28 joints using C-reactive protein (DAS28-CRP) and with the Clinical Disease Activity Index (CDAI). The Health Assessment Questionnaire (HAQ) was used as a measure of self-reported disability.

#### Imaging

##### Echocardiography

The protocol for transthoracic 2-dimensional (2D) echocardiography was detailed in prior manuscripts [10–12]. Using apical 2 and 4-chamber views, as well as real-time 3-dimensional (3D) echocardiography, left ventricular (LV) structural measures (LV mass (LVM), end-diastolic volume index (EDVI), end-systolic volume index (ESVI), and relative wall thickness (RWT)) were measured according to the most recent American Society of Echocardiography (ASE) guidelines [13]. LVM/EDV was evaluated as an alternate measure for RWT [13, 14]. LV mass (LVM) was calculated as per ASE convention [13], using LV internal diameter at end-diastole (LVIDd) and at end-systole (LVIDs) and the interventricular septal (IVSd) and posterior wall thickness (PWd) obtained in the long axis view. LVM index (LVMI) was defined as LVM indexed to height (2.7 m^2^). Baseline scans (*n* = 158) were re-read side by side with the follow-up scans (*n* = 60) by the same echocardiographer (KI) without blinding to time sequence*.*

Sex-neutral as well as sex-specific cut offs for LV structure derived from the general population were utilized: LVMI (51 g/m^2.7^ sex neutral; 50 g/m^2.7^ for men and 47 g/m^2.7^ for women) [15, 16], ESVI (31 mL/m^2^ for men and 24 mL/m^2^ for women), RWT (< 0.42) [17–20], LVM indexed to EDV (LVM/EDV) (< 1.23 in women; < 1.22 in men) [14].

##### Definitions of remodeling

LV remodeling states are defined in the general population using standardized cut-off values of LVMI and RWT. General population cut-offs for elevated LVMI are > 115 g/m^2^ for men and > 95 g/m^2^ for women, and the sex independent cut off is 51 g/ht^2.7^ and RWT > 0.42 [15]. Concentric hypertrophy (CH) is defined as LVM > 95 g/m^2^or LVMI > 115 g/m^2^ and RWT > 0.42. Concentric remodeling (CRM) is defined as LVM < 95 g/m^2^ or LVMI < 115 g/m^2^ and RWT > 0.42. Eccentric hypertrophy (EH) is defined as LVM > 95 g/m^2^ or LVMI > 115 g/m^2^ and RWT < 0.42. However, because the RA patients in this study did not have clinical HF, we utilized LVMI > 90 percentile indexed to height^2.7^ with RWT > 0.42 to define LV remodeling categories. Categories were constructed without regard to sex, as 84% of our cohort was female and there were no statistical differences in LVMI ht^2.7^ in male vs female (*p* = 0.52) within our cohort. Thus, the following cut-offs were utilized to define remodeling states in this RA cohort: (1) normal geometry, LVMI < 90 percentile and RWT < 0.42 and (2) CRM, LVMI < 90 percentile and RWT > 0.42; (3) CH, LVMI > 90 percentile and RWT > 0.42; and (4) EH, LVMI > 90 percentile and RWT < 0.42.

#### Cardiac FDG PET-CT

Cardiac PET-CT scans were performed at baseline to measure myocardial 18F-fluorodeoxyglucose (FDG) uptake, rest and stress myocardial perfusion using 13N-ammonia, and coronary artery calcium (CAC). These methods have been described extensively in prior publications [11, 12].

### Laboratory measurements

Phlebotomy was performed on the morning of each visit after an overnight fast. Details of laboratory measurements have been previously published [10]. Cholesterol (HDL, LDL, triglycerides), C-reactive protein (CRP), glucose, insulin, IL (interleukin)-6, BNP, and galectin-3 levels were measured in the Biomarkers Core Laboratory of the Columbia University Clinical and Translational Research Center. Troponin-I levels were measured by the Quanterix SimoaTM Human Troponin-I 2.0 immunoassay.

### Statistical analysis

Means and standard deviations (SDs) for normally distributed variables, and counts and percentages for categorical variables, were calculated. For continuous variables, differences were compared using Student’s *t*-tests or the Wilcoxon-Mann–Whitney test/signed ranks. Categorical variables were compared using the chi-squared goodness of fit test, Fisher’s exact test, or McNemar’s test, as appropriate. Linear regression was used to model the associations of clinical and laboratory characteristics with the primary outcomes, CRM and RWT. Univariable and multivariable models were constructed to identify variables associated with these LV structural outcomes. Multivariable models were constructed by including any variable associated with the outcome (*p* < 0.25) in univariable models, and a *p*-value of < 0.05 was considered significant for final models. All multivariable models were examined for co-linearity, omitted variables, and outliers. All analyses were performed using Stata version 16 (StataCorp, College Station, TX).

## Results

### Patient characteristics

Characteristics of the full cohort (*n* = 158) and the longitudinal subset (*n* = 60) at baseline were previously reported [10] and are re-summarized in Table 1. The longitudinal subset was largely similar to the full cohort except for shorter RA duration, higher frequency of methotrexate use, and higher mean CRP level. Statistically significant changes from baseline to follow-up in the longitudinal subset included decreases in DAS28CRP and IL-6 levels, less frequent use of methotrexate, and an increase in mean systolic blood pressure (SBP).

**Table 1:** Baseline characteristics of the RHYTHM full cohort and subset

Characteristic	Full cohort Baseline (*n* = 158)	Longitudinal Subset at baseline (*n* = 60)	Longitudinal Subset at follow-up (*n* = 60)
**Demographic**			
Age	54 ± 12	53 ± 11	57 ± 12
Female	131 (84)	49 (82)	49 (82)
Race/ethnicity	*(150)*		
White	54 (36)	24(40)	
Black	27 (18)	10 (17)	
Hispanic	62 (41.3)	24 (40)	
Other	7 (4.67)	2 (3.3)	
**RA characteristics**			
Disease duration (years)	10.7 ± 11.9	8.3 ± 9.6	ND
RF or anti-CCP (% positive)	107 (73.3)	41 (69.5)	ND
CDAI	17.5 ± 12.4	16.7 ± 12.3	13.6 ± 12
DAS28-CRP	3.71 ± 1.36	3.26 ± 1.1	3.1 ± 1.3**
CRP per mg/liter	5.1 ± 7.5	6.1 ± 9.4	7.1 ± 18.8*
IL-6 (log) per mg/liter	1.1 ± 1.2	1.2 ± 1.1	0.84 ± 1.1*
BNP per pg/mL	21.8 ± 17.8	21.7 ± 20.6	ND
Troponin-I (pg/mL)	1.08 ± 1.56	0.90 ± 1.3	ND
Galectin-3 (ng/mL)	9.56 ± 4.86	9.64 ± 5.6	ND
**RA medication**			
No DMARDs	15 (9.6)	3 (5)	8 (13)
MTX	102 (65)	46 (77)	25 (42)**
Targeted DMARDs	64 (41)	25 (42)	35 (58)
TNF inhibitor	45 (29)	19 (30)	21 (35)
Prednisone	47 (30.1)	15 (25)	4 (7.3)
**CV risk factors**			
Current smoker	16 (10.3)	6 (10)	4 (7.1)
Ever smoker	60 (38.7)	22 (36.7)	ND
SBP mm/Hg	118 ± 16.7	116 ± 16.8	124 ± 17.0**
BP medications	53 (34)	12 (20)	13 (23)
Statin	23 (14.7)	12 (20)	13 (22.8)
Total cholesterol	194 ± 37.9	187 ± 33	186.3 ± 30.8
LDL	112 ± 33.1	107.2 ± 29.9	103.6 ± 27.5
Diabetes^a^	13 (7.9)	4 (7.4)	8 (14.3)
**LV structure parameters**			
LVMI g/m^2.7^	29.2 ± 5.06	28.4 ± 4.89	28.3 ± 4.79
EDVI mL/m^2^	47.2 ± 11.0	50.4 ± 11.4	47.7 ± 10.2
ESVI mL/m^2^	17.7 ± 5.06	19.2 ± 5.66	18.9 ± 4.88
RWT	0.34 ± 0.09	0.32 ± 0.086	0.40 ± 0.087**
LVM/EDV	1.33 ± 0.24	1.24 ± 0.22	1.32 ± 0.21*
**PET/CT cardiac measures**			
**CAC score**			
**0**	106 (67.9)	44	32 (58.2)
≥ **100**	27 (17.2)	10	15 (27.3)
**Mean myocardial SUV, mean**	2.54 ± 2.04	2.7 ± 2.2	ND
**Max myocardial SUV, mean**	3.95 ± 3.29	4.5 ± 3.6	ND
**Myocardial flow reserve**	2.92 ± 0.69	2.87 ± 0.62	ND

Continuous values are expressed as mean ± SD and categorical values are expressed as *n* (%)

Italicized numbers (*n*) in parenthesis adjacent to % refer to number of patients who are reporting on that category

*ND*, not done; *LVMI*, left ventricular mass index; *EDVI*, end-diastolic volume index; *ESVI*, end-systolic volume index; *RWT*, relative wall thickness

^*^p < 0.05 **p < 0.01 comparing baseline subset (*n* = 64) vs follow-up subset (*n* = 64)

^a^Diabetes was defined as a fasting serum glucose level of ≥ 126 mg/dl or use of antidiabetic medications

### LV structure

LV structural measures are summarized in Table 1. The following mean baseline levels for the full RA cohort were statistically significantly lower than published cut-off values for the general population: mean baseline LVMI of 29.2 ± 5.06 (vs 51 g/m^2.7^ (*p* < 0.01) sex neutral; 50 g/m^2.7^ for men (*p* < 0.01) and 47 g/m^2.7^ for women (*p* < 0.01)) [15, 16]; mean baseline EDVI of 47.2 ± 11.0 (vs 74 mL/m^2^ for men (*p* < 0.01), 61 mL/m^2^ for women (*p* < 0.01)); mean baseline ESVI of 17.7 + 5.06 (vs 31 mL/m^2^ for men (*p* < 0.01) and 24 mL/m^2^ for women (*p* < 0.01)), mean baseline RWT of 0.34 ± 0.09 (vs < 0.42 (*p* < 0.01)) [17–20].

Mean baseline LVM indexed to EDV (LVM/EDV) of 1.33 ± 0.24 was above established cut-off values (< 1.23 in women; < 1.22 in men) [14]. Fifty-eight of the 60 patients enrolled in the longitudinal follow-up had complete echocardiographic data for both time points. At follow-up, both RWT and LVM/EDV increased significantly over time (*p* < 0.01 and *p* < 0.05, respectively) while mean LVMI, EDVI and ESVI were not significantly different from baseline (*p* = 0.82).

The percentages of patients fulfilling LV remodeling criteria are shown in Table 2. At baseline, 60% of the longitudinal cohort had normal geometry (normal LVMI and RWT), but only 40% retained normal geometry at follow-up. The predominant abnormal geometry at follow-up was CRM which increased significantly from 10% at baseline to 31% at follow-up. There were no significant changes in the prevalence of CH or EH from baseline to follow-up. Lastly, 4 patients with CRM at baseline reverted to normal on follow-up and 1 patient with EH at baseline reverted to normal on follow-up.

**Table 2:** Baseline and follow-up changes in LV remodeling categories

LV remodeling categories*	Baseline % (*n* = 153)	Longitudinal subset at baseline (*n* = 58) % (*n*)	Longitudinal subset at follow-up (*n* = 58) % (*n*)	Baseline subset (*n* = 53) vs. follow-up subset (*n* = 53); *p*-value**
Normal geometry	78% (119)	60% (35)	40% (23)	0.15
CRM	13% (20)	10.3% (6)	31% (18)	***0.0088***
CH	1.3% (2)	1.7% (1)	3.4% (2)	Null^***^
EH	7.8% (12)	17% (10)	15% (9)	0.48

Concentric remodeling (CRM): relative wall thickness (RWT) > 0.42 and LVMI < 90% tile

Concentric hypertrophy (CH): RWT > 0.42 and LVMI > 90% tile

Eccentric hypertrophy (EH): RWT < 0.42 and LVMI > 90% tile

^*^Normal geometry: RWT < 0.42 and LVMI < 90% tile

^**^McNemar’s test

^***^Unable to calculate *p*-value due to small numbers

### Determinants of LV remodeling

Demographic and CV risk factors and RA-associated disease variables were examined as determinants of CRM at baseline and at follow-up (other remodeling states were not examined due to small numbers). Similar analyses were performed for individual measures of wall thickness (RWT and LVM/EDV) (Tables 3 and 4; Supplementary Tables 2 and 3). In multivariable analyses with CRM as outcome, there was a trend in association of higher IL-6 levels with having CRM at follow-up (OR 2.55; 95% CI 0.99–6.58; *p* = 0.053) but not with baseline CRM (Tables 5 and  6; Supplementary Table 1). In multivariable analyses with RWT as outcome, age (*β* = 0.0043; *p* = 0.023) and tocilizumab use (*β* = 0.50; *p* = 0.043) were associated with higher baseline RWT (Table 3). In multivariable analyses with LVM/EDV as outcome, higher BMI at baseline was associated with higher LVM/EDV (*β* = 0.012; *p* = 0.010) (Supplementary Table 2). In multivariable analyses with LVMI as outcome, SBP was marginally associated with a higher likelihood of baseline 3D LVMI > 90% (OR = 1.07; 95% CI 1.02–1.12; *p* = 0.009) (Supplementary Table 4) as well as increasing RWT over time (*β* = 0.00035; *p* = 0.028) (Table 4).

**Table 3:** Univariable and multivariable regression table for baseline RWT

Baseline RWT (Log)	Univariable (*n* = 156)		Multivariable (*n* = 142)	
	***β***	***p***	***β***	***p***
**Demographics**				
Age, per year	0.0047	***0.004***	*0.0043*	***0.023***
Male versus female	0.028	0.60	…	…
Race				
White	REF	REF		
Black	0.056	0.35	…	…
Hispanic	0.010	0.83		
Other	− 0.028	0.78		
BMI, per kg/m^2^	0.0082	***0.013***	0.0033	0.37
**RA characteristics**				
RA duration (square root), per year	0.0010	0.93	…	…
AM stiffness (square root), per minute	− 0.0050	0.35	…	…
Joint deformities (square root)	− 0.0076	0.63	…	…
CDAI (square root), per unit	0.016	0.20	…	…
DAS28CRP, per unit	0.0299	***0.051***	0.026	0.10
RF > 40, units	0.0062	0.88	…	…
CCP > 250, units	− 0.034	0.42	− 0.029	0.49
Square root CRP, per mg/L	0.028	0.082	…	…
Log IL-6, per pg/mL	0.024	0.18	…	…
Log BNP, per pg/mL	− 0.00084	0.98	…	…
Log troponin-I, per pg/mL	0.049	***0.010***	…	…
Log galectin-3, per ng/mL	0.11	***0.021***	…	…
**RA medication**				
NSAID use, yes versus no	− 0.051	0.22	…	…
Prednisone use, yes versus no	0.0047	0.91	…	…
Leflunomide use, yes versus no	0.097	0.19	…	…
TNF inhibitor use, yes versus no	− 0.070	0.12	…	…
Tocilizumab use, yes versus no	0.27	0.12	*0.50*	***0.043***
**CV risk factors**				
Current smoker, yes versus no	0.054	0.41	…	…
Ever smoker, yes versus no	0.091	***0.028***	0.057	0.19
SBP, mm/Hg	0.0018	0.16	− 0.00025	0.85
Statin use, yes versus no	0.013	0.82	…	…
ASA use, yes versus no	0.0023	0.97	…	…
Total cholesterol, per mg/dl	0.000042	0.94	…	…
LDL, per mg/dl	0.00042	0.52	…	…
Square root HDL, per mg/dl	− 0.022	0.21	− 0.011	0.55
**Prob > *F***			*0.0080*	
***R***^**2**^			0.14	
**Adjusted *R***^**2**^			0.0897

**Table 4:** Univariable and multivariable regression table for annualized rate of change RWT

Annualized rate of change RWT	Univariable model (*n* = 59)		Multivariable model (*n* = 53)	
	***β***	***p***	***β***	***p***
**Demographics (baseline)**				
Age, per year	0.000072	0.74	− 0.000161	0.53
Male versus female	0.0097	0.15	0.0043	0.57
Race				
White	REF	REF	REF	REF
Black	0.0012	0.86	…	…
Hispanic	0.0067	0.21		
Other	− 0.017	0.37		
BMI, per kg/m^2^	0.00014	0.75	…	…
**RA characteristics**				
RA duration (square root), per year	0.0021	0.19	− 0.000041	0.98
Joint deformities (square root)	0.0023	0.20	…	…
DAS28CRP (baseline)	− 0.00055	0.77	…	…
DAS28CRP (follow-up)	− 0.0031	0.64	…	…
Averaged DAS28CRP (baseline + fu)	− 0.0011	0.62	…	…
CDAI (square root) (baseline)	− 0.000050	0.98	…	…
CDAI (square root) (follow-up)	− 0.00012	0.63	− 0.0027	0.12
Averaged CDAI (baseline + fu)	− 0.0013	0.38	…	…
RF (baseline) > 40, units	− 0.00499	0.32	…	…
CCP (baseline) > 250, units	− 0.0033	0.52	…	…
Square root CRP (baseline), per mg/liter	0.00047	0.78	…	…
Square root CRP (follow-up), per mg/liter	0.0010	0.41	…	…
Log IL-6, (baseline) per mg/liter	0.0018	0.41	…	…
Log IL-6, (follow-up) per mg/liter	0.0029	0.19	0.0018	0.51
Log BNP (baseline), per pg/mL	− 0.0044	0.33	…	…
Log troponin-I (baseline), per pg/mL	− 0.00032	0.89	…	…
Log galectin-3 (baseline), per ng/mL	0.0084	0.13	0.0099	0.096
**RA medications (follow-up)**				
NSAID use, yes versus no	0.0013	0.81	…	…
Prednisone use, yes versus no	− 0.0048	0.63	…	…
Hydroxychloroquine use, yes versus no	0.015	0.19	0.025	0.11
TNF inhibitors use, yes versus no	− 0.0045	0.93	…	…
Tocilizumab use, yes versus no	0.0033	0.69	…	…
**CV risk factors**				
Current smoker, yes versus no	− 0.0044	0.66	…	…
SBP (baseline), per mm/Hg	0.00023	0.11	*0.00035*	***0.028***
SBP (follow-up), per mm/Hg	0.00012	0.42	…	…
Statin use (follow-up), yes versus no	0.0048	0.42	…	…
ASA use (follow-up), yes versus no	0.011	***0.053***	0.012	0.099
Total cholesterol, per mg/dl	− 0.000018	0.82	…	…
LDL, per mg/dl	− 0.00011	0.23	…	…
HDL, per mg/dl	0.00014	0.32	…	…
**Prob > *F***	…		*0.071*	
***R***^**2**^	…		0.29	
**Adjusted *R***^**2**^	…		0.14

**Table 5:** Univariable and multivariable regression table for baseline CRM

Baseline concentric remodeling (CRM)	Univariable (*n* = 151)			Multivariable (*n* = 137)		
	**OR**	**95% CI**	***p*-value**	**OR**	**95% CI**	***p*-value**
**Demographics**						
Age, per year	*1.05*	*1.00–4.10*	***0.029***	1.04	0.98–1.099	0.21
Male versus female	1.48	0.44–4.92	0.52	1.15	0.28–4.68	0.85
Race						
White	REF	REF	0.54	REF	REF	0.36
Non-White	0.74	0.28–1.97		0.56	0.16–1.92	
BMI, per kg/m^2^	1.07	0.98–1.14	0.12	1.03	0.95–1.12	0.41
**RA characteristics**						
RA duration (square root), per year	0.87	0.64–1.18	0.37	…	…	…
Joint deformities (square root)	0.88	0.59–1.30	0.52	…	…	…
CDAI (square root), per unit	1.22	0.89–1.66	0.22	1.30	0.88–1.92	0.19
DAS28CRP, per unit	1.19	0.83–1.70	0.29	…	…	…
RF > 40, units	0.54	0.20–1.46	0.22	…	…	…
CCP > 250, units	0.61	0.23–1.61	0.32	0.84	0.27–2.65	0.77
Square root CRP, per mg/L	0.90	0.59–1.38	0.63	…	…	…
Log IL-6, per pg/mL	1.02	0.68–1.53	0.92	0.85	0.52–1.39	0.52
RBM IL-6 (binary)	0.69	0.18–2.56	0.58	…	…	…
Log BNP, per pg/mL	2.3	0.53–9.88	0.26	…	…	…
Log troponin-I, per pg/mL	1.34	0.87–2.08	0.18	…	…	…
Log galectin-3, per ng/mL	2.72	0.93 − 7.98	0.069	2.31	0.62–8.59	0.21
**RA medication**						
NSAID use, yes versus no	0.91	0.34–2.48	0.86	…	…	…
Prednisone use, yes versus no	1.43	0.52–3.90	0.49	…	…	…
Hydroxychloroquine use, yes versus no	1.92	0.56–6.50	0.30	…	…	…
Methotrexate use, yes versus no	1.46	0.50–4.33	0.49	…	…	…
TNF inhibitor use, yes versus no	0.63	0.20–2.02	0.43	…	…	…
Tocilizumab use, yes versus no	*15.3*	*1.31 − 177.78*	***0.029***	…	…	…
**CV risk factors**						
Current smoker, yes versus no	0.98	0.20–4.71	0.98	…	…	…
Ever smoker, yes versus no	2.46	0.92 − 6.53	0.072	1.91	0.65–5.60	0.24
SBP (baseline), mm/Hg	1.02	0.99–1.05	0.13	1.01	0.98–1.04	0.58
Statin use, yes versus no	0.65	0.14–3.05	0.59	…	…	…
ASA use, yes versus no	0.98	0.20–4.71	0.98	…	…	…
Total cholesterol, per mg/dl	0.99	0.98–1.01	0.46	…	…	…
LDL, per mg/dl	0.99	0.98–1.01	0.50	…	…	…
Square root HDL, per mg/dl	1.04	0.70–1.57	0.83	…	…	…
**Prob > *χ***^**2**^				0.21		
**Pseudo *R***^**2**^				0.12

**Table 6:** Univariable and multivariable regression table for follow-up CRM

Follow-up concentric remodeling (CRM)	Univariable model (*n* = 51)			Multivariable (*n* = 43)		
	**OR**	**95% CI**	***p*-value**	**OR**	**95% CI**	***p*-value**
**Demographics (baseline)**						
Age, per year	1.02	0.97–1.07	0.49	1.01	0.93–1.09	0.76
Male versus female	1.6	0.37–6.91	0.53	1.25	0.15–10.6	0.84
Race						
White	REF	REF		REF	REF	
Black	3.78	0.65–22.02	0.14	3.82	0.60–24.4	0.84
Hispanic	1.74	0.48–6.28	0.39			
Other	…	…	…			
BMI, per kg/m^2^	1.07	0.96–1.19	0.23	1.05	0.92–1.19	0.48
**RA characteristics**						
RA duration (square root), per year	1.21	0.83–1.78	0.32	…	…	…
Joint deformities (square root)	1.28	0.83–1.97	0.27	…	…	…
DAS28CRP (baseline)	1.09	0.71–1.65	0.70	…	…	…
DAS28CRP (follow-up)	1.28	0.82–2.01	0.28	…	…	…
Averaged DAS28CRP (baseline + fu)	1.24	0.76–2.05	0.39	…	…	…
CDAI (square root) (baseline)	1.08	0.75–1.57	0.67	…	…	…
CDAI (square root) (follow-up)	1.14	0.80–1.62	0.46	…	…	…
Averaged CDAI (baseline + fu)	1.01	0.96–1.07	0.65	…	…	…
RF (baseline) > 40, units	2.9	0.44–19.3	0.27	0.26	0.046–1.50	0.13
CCP (baseline) > 250, units	1.17	0.36–3.76	0.79	…	…	…
Square root CRP (baseline), per mg/liter	1.29	0.84–1.97	0.24	…	…	…
Square root CRP (follow-up), per mg/liter	0.90	0.62–1.30	0.57	…	…	…
Log IL-6 (baseline), per mg/liter	1.44	0.87–2.37	0.15	…	…	…
Log IL-6 (follow-up), per mg/liter	1.52	0.89–2.61	0.13	*2.55*	*0.99 − 6.58*	***0.053***
Log BNP (baseline), per pg/mL	1.96	0.72–5.30	0.19	1.91	0.51–7.09	0.33
Log troponin-I (baseline), per pg/mL	1.06	0.61–1.85	0.83	…	…	…
Log galectin-3 (baseline), per ng/mL	1.43	0.41–4.97	0.57	…	…	…
**RA medications (follow-up)**						
NSAID use, yes versus no	1.15	0.34–3.93	0.82	…	…	…
Prednisone use, yes versus no	0.79	0.067–9.44	0.85	…	…	…
Hydroxychloroquine use, yes versus no	3.87	0.33–46.05	0.28	…	…	…
TNF inhibitors use, yes versus no	0.83	0.25–2.80	0.77	…	…	…
Tocilizumab use, yes versus no	5.8	0.55–60.7	0.14	…	…	…
**CV risk factors**						
Current smoker, yes versus no	0.79	0.067–9.44	0.85	…	…	…
SBP (baseline), per mm/Hg	0.998	0.96–1.03	0.87	…	…	…
SBP (follow-up), per mm/Hg	0.99	0.96–1.03	0.71	…	…	…
Statin use (follow-up), yes versus no	1.54	0.39–6.03	0.54	…	…	…
ASA use (follow-up), yes versus no	*4.14*	*1.00 − 17.05*	***0.049***	4.01	0.46–34.89	0.21
Total cholesterol (follow-up), per mg/dl	1.00	0.98–1.03	0.58	…	…	…
LDL (follow-up), per mg/dl	1.00	0.98–1.02	0.99	…	…	…
HDL (follow-up), per mg/dl	0.998	0.97–1.03	0.93	…	…	…
**Prob > *χ***^**2**^				0.0597		
**Pseudo *R***^**2**^				0.26		

There were no significant associations (univariable or multivariable analyses) between measures of myocardial inflammation and perfusion vs baseline/follow-up LV structure, including CRM and RWT. Additionally, there were no significant associations (univariable or multivariable analyses) between BNP, troponin-I, and galectin-3 and baseline/follow-up LV structure, including CRM and RWT.

## Discussion

LV structure is assessed by LVM and RWT on two or three-dimensional TTE. Several proxy measures for RWT exist, including LVM indexed to EDV dimensions. Generally, increased LVM and RWT indicate abnormal LV geometry.

In this study, we confirmed that CRM, defined by increased RWT but normal LVM, is prevalent in RA patients without HF and, importantly, showed for the first time that the prevalence increases over time. Furthermore, our data suggest several inflammatory determinants of LV remodeling and its progression including higher IL-6 levels or use of an IL-6 inhibitor (tocilizumab), as well as demographic and CV determinants including age, BMI, and SBP.

In our prospective sub-cohort, CRM was the most prevalent form of remodeling at baseline (13%) and increased significantly to 31% on follow-up. RWT and LVM/EDV also both increased significantly over time (*p* < 0.05 for both), consistent with the overall increasing prevalence of CRM. Other individual LV structural parameters such as LVMI, EDVI, and ESVI did not change significantly on follow-up.

Our findings are in agreement with prior cross-sectional RA studies which also demonstrate that concentric geometry is the most prevalent remodeling form [ 8, 9] . This is notable as concentric geometry (remodeling and/or hypertrophy) has been linked to increased CV events including HF in the general population [21–23]. Whether this increase in CRM in RA is a risk factor for future clinical HF needs to be explored in prospective studies. Several other prospective RA studies reported statistically significant *declines* in LVMI, wall thickness, EDV, and ESV in RA patients without clinical HF [24], diverging from our results. This could be attributable to differences in demographics, RA disease features (duration, disease activity, treatment), sample size, conventional CV risk factors, or other variables.

Beyond the usual demographic and CV variables discussed above, higher rates of LV remodeling in RA vs non-RA controls have been hypothesized to be due to the higher levels of inflammation in RA. Indeed, we observed an association between inflammatory measures- IL6- and LV structure. Namely, every logged unit (mg/L) increase in IL-6 was associated with 2.5 times higher odds of CRM on follow-up. These findings are consistent with the hypothesis that higher levels of IL-6 induce adverse LV structure/geometry, likely mediated by activation of pro-fibrotic and myocyte hypertrophy pathways [25–27]. Tocilizumab use at baseline was also associated with higher baseline RWT. As use of anti-inflammatory medications, particularly biologic DMARDs in RA, is associated with higher burden of inflammation, this could be considered a marker of those with severe RA disease and possibly LV remodeling.

In as far as the association between tocilizumab and IL-6 levels are concerned, given the small numbers of patients on tocilizumab at baseline (*n* = 3) and on follow-up (*n* = 6), meaningful statistical differences in IL-6 levels could not be inferred. However, IL-6 levels were consistently higher in those on tocilizumab vs those not on tocilizumab (results not shown), which may reflect an accumulation of free/unmetabolized IL-6 in circulation resulting from uptake of IL-6 receptors after tocilizumab treatment.

In our multivariable analyses, older age at baseline was associated with higher baseline RWT, which parallels findings in both RA [7, 28] and non-RA studies [29–31]. This reinforces the idea that aging remains an independent risk factor for LV remodeling.

Additionally, baseline BMI was associated with adverse remodeling/geometry, including higher baseline LVM/EDV, and baseline LVMI > 90%. This is in line with at least one RA study in which BMI was significantly associated with higher baseline LVMI [7]. It is also consistent with an array of non-RA studies which demonstrated BMI independently associating with higher LV mass [29, 31–33].

Baseline SBP was associated with baseline LVMI > 90% as well as increasing RWT over time. While many studies in non-RA patients demonstrate significant associations between SBP and higher/increasing LVMI [29, 32], the majority of RA studies do not support this association. In our current study, the prevalence of hypertension was low (mean SBP = 117.7; 1/3 on BP medications); therefore, any possible associations between BP and LV structure could have been attenuated.

There were no associations with troponin-I and BNP and LV structure. Given that both are released as a response to myocyte injury/necrosis, it is possible that our patients were too early in the course of myocardial/LV damage and remodeling to be able to detect these markers.

Limitations of this study include the absence of non-RA controls, which does not allow for direct comparisons and establish whether such changes in LV structure are unique/specific to RA. While only a limited number of patients returned for follow-up (about 1/3 of baseline cohort), we were still able to detect significant changes in key parameters of LV structure.

## Conclusion

In conclusion, LV remodeling is prevalent in RA patients without clinical HF and increases to 60% over time. Adverse LV structure/remodeling at baseline and follow-up are associated with higher IL-6 levels and use of tocilizumab. These findings suggest that inflammatory cytokines such as IL-6 could serve as biomarkers for LV remodeling in RA patients and possibly future development of HF. Investigating whether LV remodeling leads to clinical HF in RA patients and whether LV remodeling is reversible or attenuated by IL-6 inhibition (tocilizumab) remain key future research objectives, ultimately to reduce the twofold higher HF mortality rate associated with RA vs non-RA [34].

## Supplementary Information

**Additional file 1: Table S1.** Follow-up CRM *excluded to those who did not have CRM at baseline*. **Table S2.** Baseline LVM/EDV**.** **Table S3.** Annualized Rate of Change in LVM/EDV. **Table S4.** Baseline 3D LVMI ht2.7 > 90%. **Table S5.** Annualized Rate of Change in LVMI2.7

## Data Availability

The datasets used and analyzed during the current study are included in this published article (and its supplementary information files) or otherwise available from the corresponding author on request.

## Abbreviations

2D: 2-Dimensional
3D: 3-Dimensional
ASE: American Society of Echocardiography
BMI: Body mass index
CAC: Coronary artery calcium
CDAI: Clinical Disease Activity Index
CH: Concentric hypertrophy
CRM: Concentric remodeling
CRP: C-reactive protein
CV: Cardiovascular
CVD: Cardiovascular disease
DAS28-CRP: Disease Activity Score for 28 joints using CRP
EDVI: End-diastolic volume index
EH: Eccentric hypertrophy
ESVI: End-systolic volume index
FDG: Myocardial 18F-fluorodeoxyglucose
HAQ: Health Assessment Questionnaire
HF: Heart failure
HFpEF: HF with preserved ejection fraction
HFrEF: HF with reduced ejection fraction
IL-6: Interleukin-6
IVSd: Interventricular septal
LDL: Low-density lipoprotein
LV: Left ventricular
LVIDs and LVIDs: LV internal diameter at end-diastole and end-systole
LVMI: LV mass index
PWd: Posterior wall thickness
RA: Rheumatoid arthritis
RHYTHM: RHeumatoid Arthritis studY of THe Myocardium
RWT: Relative wall thickness
SBP: Systolic blood pressure
SD: Standard deviation
SUV: Standardized uptake Value
