# Supplementary material for: Left ventricular remodeling in rheumatoid arthritis patients without clinical heart failure
Source: Arthritis Res Ther. 2023 Jul 21;25:124. doi: 10.1186/s13075-023-03113-8 (PMC10362590; doi:10.1186/s13075-023-03113-8)
Supplement: Supplementary file 1 — Additional file 1: Table S1. Follow-up CRM *excluded to those who did not have CRM at baseline*. Table S2. Baseline LVM/EDV. Table S3. Annualized Rate of Change in LVM/EDV. Table S4. Baseline 3D LVMI ht2.7 > 90%. Table S5. Annualized Rate of Change in LVMI2.7 [file 13075_2023_3113_MOESM1_ESM.docx]

| **Table 1. Follow-up CRM *excluded to those who did not have CRM at baseline*** | | | | | | |
| --- | --- | --- | --- | --- | --- | --- |
| **Follow-Up Concentric Remodeling (CRM)** | **Univariable Model (n=46)**  **OR 95% CI P value** | | | **Multivariable (n=45)**  **OR 95% CI P-value** | | |
| **Demographics (baseline)** | | | | | | |
| Age, per year | 1.02 | 0.97-1.07 | 0.40 | 1.00 | 0.93-1.08 | 0.88 |
| Male versus female | 1.34 | 0.26-6.86 | 0.73 | 1.35 | 0.19-9.61 | 0.76 |
| Race  White  Black  Hispanic  Other | REF  **6.40**  2.33  … | REF  **0.89-45.99**  0.60-9.03  … | **0.065**  0.22  … | REF  **8.07**  2.46  … | REF  **0.78-83.00**  0.38-15.75  … | **0.079**  0.34  … |
| BMI, per kg/m^2^ | 1.07 | 0.96-1.19 | 0.23 | 1.06 | 0.93-1.22 | 0.37 |
| **RA Characteristics** | | | | | | |
| RA Duration (square root), per year | 1.28 | 0.85-1.92 | 0.24 | 1.20 | 0.69-2.08 | 0.52 |
| Joint deformities (square root) | 1.23 | 0.78-1.95 | 0.37 | … | … | … |
| DAS28CRP (baseline) | 1.15 | 0.74-1.77 | 0.53 | … | … | … |
| DAS28CRP (follow-up) | 1.33 | 0.81-2.18 | 0.285 | … | … | … |
| Averaged DAS28CRP (baseline + fu) | 1.32 | 0.77-2.26 | 0.31 | 0.83 | 0.36-1.91 | 0.66 |
| CDAI (square root) (baseline) | 1.12 | 0.76-1.64 | 0.58 | … | … | … |
| CDAI (square root) (follow-up) | 1.14 | 0.78-1.66 | 0.49 | … | … | … |
| Averaged CDAI (baseline + fu) | 1.01 | 0.96-1.07 | 0.65 | … | … | … |
| RF (baseline) >40, units | 0.77 | 0.23-2.63 | 0.68 | … | … | … |
| CCP (baseline) >250, units | 1.14 | 0.33-3.90 | 0.83 | … | … | … |
| Square root CRP (baseline), per mg/liter | 1.36 | 0.87-2.14 | 0.17 | … | … | … |
| Square root CRP (follow-up), per mg/liter | 1.21 | 0.67-2.17 | 0.52 | … | … | … |
| Log IL-6 (baseline), per mg/liter | 1.42 | 0.86-2.36 | 0.17 | … | … | … |
| Log IL-6 (follow-up), per mg/liter | ***1.77*** | ***0.94-3.35*** | ***0.078*** | 1.61 | 0.75-3.47 | 0.22 |
| Log BNP (baseline), per pg/mL | 1.68 | 0.58-4.82 | 0.34 | … | … | … |
| Log troponin-I (baseline), per pg/mL | 1.01 | 0.57-1.78 | 0.98 | … | … | … |
| Log galectin-3 (baseline), per ng/mL | 1.91 | 0.49-7332 | 0.34 | … | … | … |
| **RA Medications (follow-up)** | | | | | | |
| NSAID use, yes versus no | 0.92 | 0.25-3.42 | 0.91 | … | … | … |
| Prednisone use, yes versus no | 0.72 | 0.060-8.61 | 0.79 | … | … | … |
| Hydroxychloroquine use, yes versus no | 3.6 | 0.30-43.08 | 0.31 | … | … | … |
| TNF inhibitors use, yes versus no | 0.55 | 0.15-2.01 | 0.37 | … | … | … |
| Tocilizumab use, yes versus no | 5.36 | 0.51-56.5 | 0.16 | … | … | … |
| **CV Risk Factors** | | | | | | |
| Current smoker, yes versus no | 0.81 | 0.068-9.70 | 0.87 | … | … | … |
| SBP (baseline), per mm/Hg | 1.00 | 0.96-1.04 | 0.96 | … | … | … |
| SBP (follow-up), per mm/Hg | 0.99 | 0.96-1.03 | 0.74 | … | … | … |
| Statin use (follow-up), yes versus no | 1.53 | 0.38-6.07 | 0.55 | … | … | … |
| ASA use (follow-up), yes versus no | 3.27 | 0.76-13.99 | 0.11 | 2.30 | 0.25-20.99 | 0.46 |
| Total cholesterol (follow-up), per mg/dl | 1.00 | 0.98-1.02 | 0.72 | … | … | … |
| LDL (follow-up), per mg/dl | 0.997 | 0.97-1.02 | 0.79 | … | … | … |
| HDL (follow-up), per mg/dl | 1.00 | 0.97-1.03 | 0.94 | … | … | … |
|  | | | | | | |
| **Prob >Chi^2^** |  | | | 0.32 | | |
| **Pseudo R^2^** |  | | | 0.17 | | |

**Table 2. Baseline LVM/EDV**

| **Baseline LVM/EDV** | **Univariable (n=154)**  **β P** | | **Multivariable (n=98)**  **β P** | |
| --- | --- | --- | --- | --- |
| **Demographics** |  |  |  |  |
| Age, per year | **0.0035** | ***0.027*** | 0.0021 | 0.28 |
| Male versus female | 0.0058 | 0.91 | … | … |
| Race  White  Black  Hispanic  Other | REF  0.083  0.060  0.15 | REF  0.15  0.19  0.16 | REF  0.060 | REF  0.29 |
| BMI, per kg/m^2^ | **0.012** | ***<0.01*** | ***0.012*** | ***0.010*** |
| **RA Characteristics** | | | | |
| RA duration (square root), per year | 0.0097 | 0.41 | … | … |
| AM stiffness (square root), per minute | -0.0051 | 0.33 | … | … |
| Joint deformities (square root) | -0.011 | 0.45 | … | … |
| CDAI (square root), per unit | 0.014 | 0.24 | -0.010 | 0.52 |
| DAS28CRP, per unit | **0.025** | **0.092** | … | … |
| RF>40, units | 0.064 | 0.11 | … | … |
| CCP>250, units | 0.017 | 0.67 | … | … |
| Square root CRP, per mg/L | 0.013 | 0.42 | … | … |
| Log IL-6, per pg/mL | 0.012 | 0.48 | … | … |
| Log BNP, per pg/mL | **-0.067** | **0.090** | -0.060 | 0.12 |
| Log troponin-I, per pg/mL | 0.022 | 0.22 | … | … |
| Log galectin-3, per ng/mL | 0.053 | 0.25 | … | … |
| **RA Medications** | | | | |
| NSAID use, yes versus no | -0.0020 | 0.96 | … | … |
| Prednisone use, yes versus no | -0.0073 | 0.86 | … | … |
| Sulfasalazine use, yes versus no | 0.085 | 0.23 | **0.15** | **0.071** |
| TNF inhibitor use, yes versus no | -0.017 | 0.69 | … | … |
| Tocilizumab use, yes versus no | 0.18 | 0.21 | … | … |
| **CV Risk Factors** | | | | |
| Current smoker, yes versus no | ***0.16*** | ***0.012*** | 0.051 | 0.52 |
| Ever smoker, yes versus no | ***0.10*** | ***0.010*** | … | … |
| SBP (baseline), mm/Hg | ***0.0026*** | ***0.027*** | 0.0014 | 0.31 |
| Statin use, yes versus no | 0.021 | 0.70 | … | … |
| ASA use, yes versus no | 0.091 | 0.16 | 0.063 | 0.39 |
| Total cholesterol, per mg/dl | -0.000054 | 0.92 | … | … |
| LDL, per mg/dl | 0.00059 | 0.33 | … | … |
| Square root HDL, per mg/dl | ***-0.049*** | ***0.004*** | 0.0054 | 0.98 |
| **Prob>F** |  | | ***0.0075*** | |
| **R-Squared** |  | | 0.23 | |
| **Adjusted R-Squared** |  | | 0.14 | |

**Table 3. Annualized Rate of Change in LVM/EDV**

|  | **Univariable Model (n=57)**  **β P** | | **Multivariable (n=43)**  **β P** | |
| --- | --- | --- | --- | --- |
| **Demographics (baseline)**    Age, per year | -0.00029 | 0.73 | -0.012 | 0.24 |
| Male versus female | 0.030 | 0.24 | 0.046 | 0.14 |
| Race  White  Black  Hispanic  Other | REF  **-0.048**  -0.024  ***-0.11*** | REF  **0.090**  0.24  ***0.038*** | REF  -0.032 | REF  0.15 |
| BMI, per kg/m^2^ (follow-up) | 0.00080 | 0.56 | 0.00097 | 0.52 |
| **RA Characteristics**  RA Duration (square root), per year | -0.0015 | 0.82 | … | … |
| Joint deformities (square root) | 0.0087 | 0.24 | … | … |
| DAS28CRP (baseline) | 0.0041 | 0.58 | … | … |
| DAS28CRP (follow-up) | -0.0097 | 0.18 | … | … |
| Averaged DAS28CRP (baseline + fu) | -0.0050 | 0.55 | … | … |
| CDAI (square root) (baseline) | 0.0072 | 0.26 | … | … |
| CDAI (square root) (follow-up) | -0.0079 | 0.17 | -0.0036 | 0.59 |
| Averaged CDAI (baseline + fu) | 0.000049 | 0.96 | … | … |
| RF (baseline)>40, units | -0.0197 | 0.33 | … | … |
| CCP (baseline)>250, units | 0.0048 | 0.81 | 0.0145 | 0.57 |
| Square root CRP (baseline), per mg/liter | -0.0029 | 0.68 | … | … |
| Square root CRP (follow-up), per mg/liter | -0.0045 | 0.41 | … | … |
| Log IL-6, (baseline) per mg/liter | 0.0081 | 0.35 | … | … |
| Log IL-6, (follow-up) per mg/liter | -0.0015 | 0.86 | … | … |
| Log BNP (baseline), per pg/mL | 0.014 | 0.36 | … | … |
| Log troponin-I (baseline), per pg/mL | -0.0054 | 0.58 | … | … |
| Log galectin-3 (baseline), per ng/mL | -0.028 | 0.19 | … | … |
| **RA Medication (follow-up)** |  |  |  |  |
| NSAID use, yes versus no | -0.029 | 0.17 | … | … |
| Prednisone use, yes versus no | -0.010 | 0.81 | … | … |
| HCQ use, yes versus no | 0.061 | 0.16 | … | … |
| Methotrexate use, yes versus no | 0.021 | 0.29 | … | … |
| TNF inhibitors use, yes versus no | 0.012 | 0.54 | … | … |
| Tocilizumab use, yes versus no | -0.021 | 0.56 | … | … |
| **CV Risk Factors** |  |  |  |  |
| Current smoker, yes versus no | -0.068 | 0.11 | -0.023 | 0.57 |
| SBP (baseline), per mm/Hg | -0.00040 | 0.51 | … | … |
| SBP (follow-up), per mm/Hg | -0.00092 | 0.12 | -0.00082 | 0.20 |
| Statin use, yes versus no | **0.041** | **0.077** | ***0.062*** | ***0.032*** |
| ASA use, yes versus no | 0.0057 | 0.81 | … | … |
| Total cholesterol, per mg/dl | 5.85e-06 | 0.99 | … | … |
| LDL, per mg/dl | -0.00012 | 0.74 | … | … |
| HDL, per mg/dl | 0.00015 | 0.78 | … | … |
| **Prob>F** |  | | 0.22 | |
| **R-Squared** |  | | 0.28 | |
| **Adjusted R-Squared** |  | | **0.082** | |

**Table 4. Baseline 3D LVMI ht^2.7^ >90%**

| **3D LVMI ht^2.7^>90%** | **Univariable (n=154)** | | | **Multivariable (n=135)** | | | |
| --- | --- | --- | --- | --- | --- | --- | --- |
|  | **OR** | **95% CI** | **P** | **OR** | **95% CI** | **P** |  |
| **Demographics (baseline)**  Age, per year | 1.02 | 0.97-1.06 | 0.44 | 0.93 | 0.85-1.02 | 0.14 |  |
| Male versus female | 1.38 | 0.36-5.31 | 0.64 | 1.24 | 0.11-13.93 | 0.86 |  |
| Race  White  Black  Hispanic  Other | REF  1.53  1.85  … | REF  0.32-7.39  0.52-6.53  … | REF  0.60  0.34  … | REF  **0.15** | REF  **0.019-1.24** | REF  **0.079** |  |
| BMI, per kg/m^2^ | ***1.093*** | ***1.01-1.18*** | ***0.030*** | **1.13** | **0.99-1.29** | **0.061** |  |
| **RA Factors** |  |  |  |  |  |  |  |
| RA duration (square root), per year | 1.27 | 0.94-1.71 | 0.12 | 0.997 | 0.63-1.56 | 0.99 |  |
| AM stiffness (square root), per min | **1.13** | **0.99-1.28** | ***0.075*** | … | … | … |  |
| Swollen joints (square root) (0-28) | 1.54 | 0.90-2.64 | 0.11 | … | … | … |  |
| Tender joints (square root) (0-28) | **1.38** | **0.93-2.04** | **0.11** | … | … | … |  |
| Joint deformities (square root) | 1.01 | 0.67-1.53 | 0.95 | … | … | … |  |
| Nodules (yes versus no) | **4.62** | **1.24-17.17** | ***0.022*** | … | … | … |  |
| CDAI (square root), per unit | ***1.48*** | ***1.02-2.14*** | ***0.038*** | ***1.80*** | ***0.99-3.25*** | ***0.053*** |  |
| DAS28CRP, per unit | ***1.54*** | ***1.02-2.343*** | ***0.041*** | … | … | … |  |
| RF>40, units | 2.16 | 0.70-6.69 | 0.18 | … | … | … |  |
| CCP>250, units | **2.79** | **0.84-9.24** | **0.092** | **5.13** | **0.86-30.43** | **0.072** |  |
| HAQ (square root) score, per unit | 1.52 | 0.46-4.95 | 0.49 | … | … | … |  |
| **RA Medications** |  |  |  |  |  |  |  |
| NSAID use, yes versus no | 1.43 | 0.49-4.17 | 0.51 | … | … | … |  |
| Prednisone use, yes versus no | 1.62 | 0.54-4.84 | 0.39 | … | … | … |  |
| Hydroxychloroquine use, yes versus no | 0.44 | 0.055-3.57 | 0.44 | … | … | … |  |
| Leflunomide use, yes versus no | 2.19 | 0.43-11.22 | 0.35 | … | … | … |  |
| Methotrexate use, yes versus no | 0.76 | 0.25-2.26 | 0.62 | … | … | … |  |
| Sulfasalazine use, yes versus no | **3.17** | **0.77-13.13** | **0.11** | … | … | … |  |
| Abatacept use, yes versus no | 0.91 | 0.11-7.62 | 0.93 | … | … | … |  |
| TNF inhibitor use, yes versus no | 0.88 | 0.26-2.93 | 0.84 | … | … | … |  |
| Log IL-6, per pg/mL | ***1.72*** | ***1.14-2.61*** | ***0.010*** | ***1.78*** | ***1.01-3.15*** | ***0.047*** |  |
| Log BNP, per pg/mL | 0.59 | 0.15-2.21 | 0.43 | … | … | … |  |
| Log troponin-I, per pg/mL | **1.74** | **1.05-3.17** | ***0.034*** | 1.77 | 0.85-3.72 | 0.13 |  |
| Log galectin-3, per ng/mL  **CV Risk Factors** | 1.76 | 0.54-5.76 | 0.35 | … | … | … |  |
| Current smoker, yes versus no | 2.38 | 0.95-9.56 | 0.22 | 4.27 | 0.64-28.25 | 0.13 |  |
| Ever smoker, yes versus no | 1.06 | 0.35-3.14 | 0.92 | … | … | … |  |
| SBP (baseline), mm/Hg | 1.03 | 1.00-1.07 | 0.028 | ***1.07*** | ***1.02-1.12*** | ***0.009*** |  |
| DBP (baseline), mm/Hg | 1.05 | 0.99-1.12 | 0.11 | … | … | … |  |
| Statin use, yes versus no | 1.46 | 0.38-5.65 | 0.58 | … | … | … |  |
| ASA use, yes versus no | 2.38 | 0.59-9.56 | 0.22 | 1.48 | 0.22-10.05 | 0.69 |  |
| Total cholesterol, per mg/dl | 1.01 | 0.99-1.02 | 0.29 | … | … | … |  |
| LDL, per mg/dl | 1.01 | 0.99-1.02 | 0.45 | … | … | … |  |
| Square root HDL, per mg/dl | 1.24 | 0.79-1.94 | 0.35 | … | … | … |  |
|  |  |  |  |  |  |  |  |
|  |  |  |  |  |  |  |  |
| **Prob>F** | … | | | ***0.0025*** | | | |
| **PSEUDO Squared** | … | | | 0.35 | | | |

**Table 5. Annualized Rate of Change in LVMI^2.7^**

|  | **Univariable Model (n=56)** | | **Multivariable (n=51)** | |
| --- | --- | --- | --- | --- |
|  | **β** | **P** | **β** | **P** |
| **Demographics (baseline)**  Age, per year | 0.0013 | 0.90 | -0.0033 | 0.78 |
| Male versus female | 0.045 | 0.89 | 0.34 | 0.36 |
| Race  White  Black  Hispanic  Other | REF  0.10  -0.27  -0.64 | REF  0.78  0.34  0.51 | REF  -0.44  (non-white) | REF  0.13 |
| BMI, per kg/m^2^ | -0.00036 | 0.99 | 0.0063 | 0.78 |
| **RA Characteristics** | | |  |  |
| RA Duration (square root), per year | -0.070 | 0.41 |  |  |
| AM stiffness (square root), per minute | -0.0075 | 0.83 |  |  |
| Joint deformities (square root) | -0.16 | 0.10 | -0.029 | 0.79 |
| DAS28CRP (baseline) | -0.056 | 0.56 |  |  |
| DAS28CRP (follow-up) | -0.073 | 0.43 |  |  |
| Averaged DAS28CRP (baseline + fu) | -0.051 | 0.62 |  |  |
| CDAI (square root) (baseline) | -0.057 | 0.49 |  |  |
| CDAI (square root) (follow-up) | -0.088 | 0.23 |  |  |
| Averaged CDAI (baseline + fu) | -0.0017 | 0.88 | 0.014 | 0.32 |
| RF (baseline)>40, units |  |  |  |  |
| CCP (baseline)>250, units |  |  | 0.35 | 0.20 |
| Square root CRP, per mg/liter | -0.064 | 0.36 |  |  |
| Log IL-6 (FU), per mg/liter | ***-0.31*** | ***0.005*** | ***-0.28*** | ***0.033*** |
| Log BNP (baseline), per pg/mL | 0.11 | 0.54 |  |  |
| Log troponin-I (baseline), per pg/mL | -0.20 | 0.12 |  |  |
| Log galectin-3 (baseline), per ng/mL | -0.10 | 0.72 |  |  |
| HAQ (square root) (baseline) score, per unit | -0.27 | 0.31 |  |  |
| **RA Medication** | | |  |  |
| NSAID use, yes versus no | -0.036 | 0.90 |  |  |
| Prednisone use, yes versus no | -0.59 | 0.31 |  |  |
| Methotrexate use, yes versus no | -0.14 | 0.61 |  |  |
| SSZ use, yes versus no | 0.67 | 0.17 |  |  |
| Abatacept use, yes versus no | 0.68 | 0.23 |  |  |
| TNF inhibitors use, yes versus no | -0.18 | 0.51 |  |  |
| TNF inhibitor use (bl), yes versus no | ***-0.52*** | ***0.055*** | ***-0.61*** | ***0.025*** |
| Tocilizumab use, yes versus no | -0.28 | 0.58 |  |  |
| **CV Risk Factors** | | |  |  |
| Current smoker (baseline), yes versus no | ***-1.20*** | ***0.019*** | -0.35 | 0.45 |
| SBP (baseline), per mm/Hg | 0.00057 | 0.94 |  |  |
| SBP (follow-up), per mm/Hg | 0.0064 | 0.39 |  |  |
| DBP (baseline), per mm/Hg | 0.011 | 0.49 |  |  |
| Statin use, yes versus no | 0.10 | 0.72 |  |  |
| ASA use, yes versus no | 0.082 | 0.78 |  |  |
| Total cholesterol, per mg/dl | -0.0017 | 0.70 |  |  |
| LDL, per mg/dl | 0.00032 | 0.94 |  |  |
| HDL, per mg/dl | -0.0050 | 0.49 |  |  |
| **Prob>F** |  | | 0.20 | |
| **R-Squared** |  | | 0.27 | |
| **Adj R-squared** |  | | 0.078 | |
